# Pharmacologic interventions for painful diabetic neuropathy: an umbrella systematic review and comparative effectiveness network meta-analysis (Protocol)

**DOI:** 10.1186/2046-4053-1-61

**Published:** 2012-12-02

**Authors:** Marcio L Griebeler, Apostolos Tsapas, Juan P Brito, Zhen Wang, Olivia J Phung, Victor M Montori, M Hassan Murad

**Affiliations:** 1 Division of Diabetes, Endocrinology, Metabolism and Nutrition, Department of Medicine, Mayo Clinic, Rochester, MN 55905, USA; 2 Second Medical Department, Aristotle University Thessaloniki, Thessaloniki, Greece; 3 Knowledge and Evaluation Research Unit, Mayo Clinic, Rochester, MN, 55905, USA; 4 Western University of Health Sciences, College of Pharmacy and Western Diabetes Institute, Rochester, MN, USA; 5 Division of Preventive Medicine, Department of Medicine, Mayo Clinic, Rochester, MN, 55905, USA

**Keywords:** Comparative effectiveness research, Diabetic neuropathy, Network meta-analysis, Systematic review

## Abstract

**Background:**

Neuropathic pain can reduce the quality of life and independence of 30% to 50% of patients with diabetes. The comparative effectiveness of analgesics for patients with diabetic neuropathy remains unclear. The aim of the current work, therefore, was to summarize the evidence about the analgesic effectiveness of the most common oral and topical agents used for the treatment of peripheral diabetic neuropathy.

**Methods:**

We will use an umbrella approach (systematic review of systematic reviews) to identify eligible randomized controlled trials (RCTs) for the most common oral or topical analgesics for painful diabetic neuropathy. Two reviewers will independently determine RCT eligibility. Disagreement will be solved by consensus and arbitrated by a third reviewer. We will extract descriptive, methodological and efficacy data in duplicate. Results will be pooled and analyzed using classic random-effects meta-analyses and network meta-analyses to compute the absolute and relative efficacy of therapeutic options. We will use the *I*^2^ statistic and Cochran’s Q test to assess heterogeneity. Risk of bias and publication bias, if appropriate, will be evaluated, as well as overall strength of the evidence.

**Discussion:**

This network meta-analysis aims to synthesize available direct and indirect evidence of effectiveness of analgesics in the treatment of painful diabetic neuropathy. The network approach will offer the opportunity to generate a ranking based on efficacy and along with known side effects, costs, and administration burdens will enable patients and clinicians to make choices that best reflect their preferences for treatment of painful diabetic neuropathy.

## Background

Diabetic neuropathy is the most common complication of diabetes. It usually has a distal symmetric pattern that slowly progresses proximally with major implications to quality of life. It affects the function, mood and sleep patterns of the majority of affected patients. Symptoms include neuropathic pain, burning, tingling, decreased sensation, and loss of temperature perception [1-4]. Neuropathic pain is present in 30% to 50% of patients with diabetes [5,6].

Glycemic control has been shown to be effective in slowing the progression of diabetic neuropathy [7]. Patients with painful neuropathy often need other agents to palliate their symptoms. Agents used include tricyclic antidepressants, anticonvulsants, serotonin-norepinephrine reuptake inhibitors, opiates, opiate-like substances and topical medications. However, these medications usually at best provide only partial pain relief [2,5,6].

There is no clear evidence-based guidance about treatment selection of analgesic agents for painful diabetic neuropathy. Stepwise approach and algorithms may be used, but comparative effectiveness of treatments to control pain in patients with diabetic neuropathy is unclear and there is no evidence of relative superiority across the different drug classes or the individual agents.

We therefore decided to conduct a systematic review to appraise and summarize the totality of evidence regarding the efficacy of the most common non-parenteral (oral and topical) analgesic therapeutic options for the treatment of diabetic neuropathy.

## Methods

### Objectives

**Figure 1:**
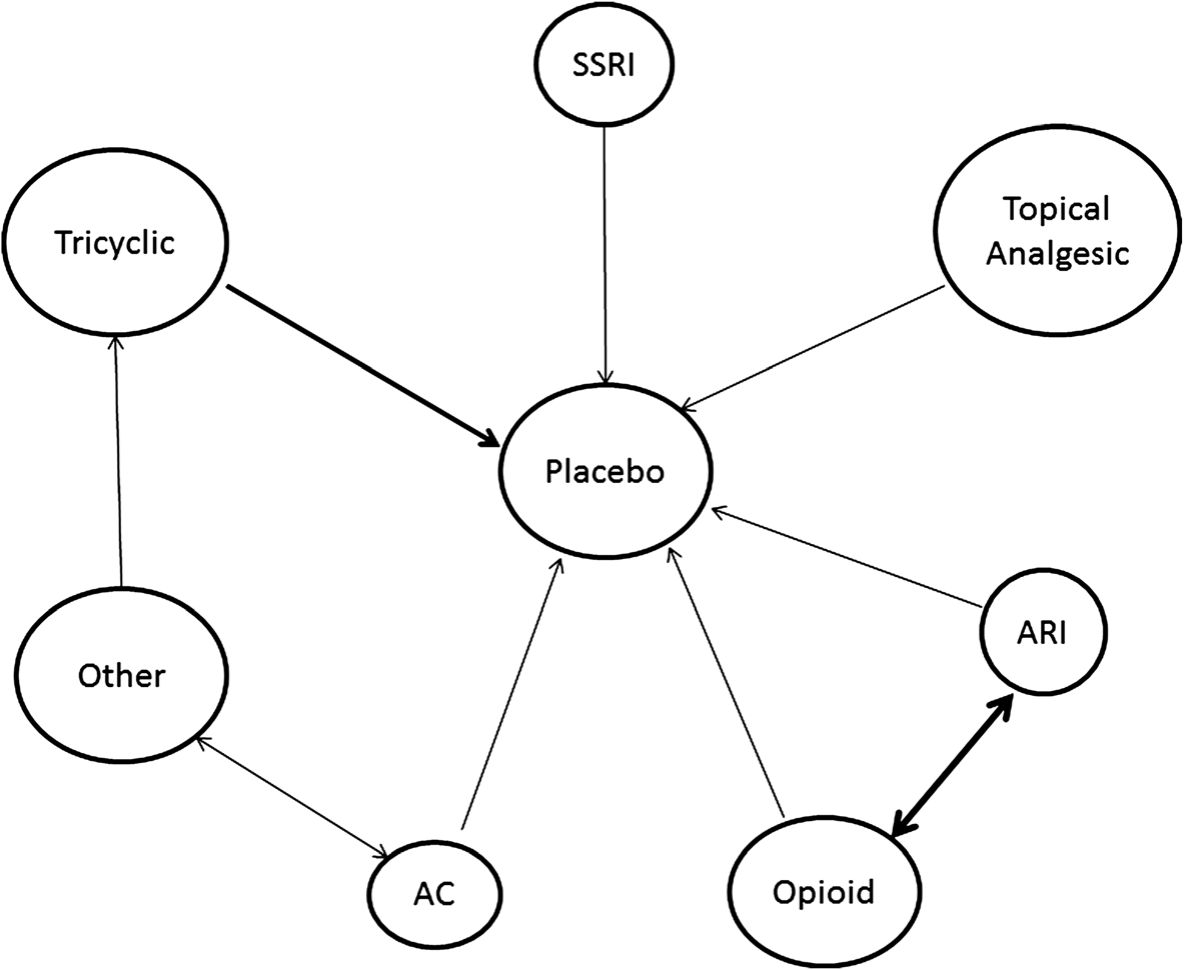
**Example of network of direct comparisons for the multiple-treatments meta-analysis for the outcome of interest that will be used.** The thickness of the connecting lines is proportional to the number of available direct comparisons. The size of each node is proportional to the number of trials that investigated each treatment. The information in this figure is preliminary and will be updated after the search and analysis. AC = anticonvulsant; ARI = aldose reductase inhibitor; SSRI = selective serotonin reuptake inhibitor.

Considering the large number of pharmacologic therapies and the availability of multiple systematic reviews that identified the efficacy trials of these drugs, we decided to follow an ‘umbrella’ [8,9] approach to identify eligible randomized controlled trials (RCTs). In brief, we will identify systematic reviews that compare available therapeutic options for painful diabetic neuropathy to placebo or any other active comparators. Eligible systematic reviews will be retrieved and used to identify relevant RCTs. We will subsequently build a dataset comprising eligible RCTs to obtain, critically appraise for quality, and extract data from each RCT to be used for the meta-analyses of relevant outcomes. Extracted data will be combined using a network design to draw inferences about the absolute and relative efficacy (from direct and indirect comparisons) of individual therapeutic options (Figure 1).

### Umbrella systematic review

#### Interventions

Two study investigators with expertise in diabetes (VMM and AT) provided input about the most common drugs used for diabetic neuropathy in USA and Europe. A list describing the most commonly used therapies deemed eligible for this review, with their minimum effective dose and therapeutic range based on American Diabetes Association (ADA) recommendations or expert opinion is presented in Table 1. For a trial to be included, the intervention dose evaluated must be at least the minimum effective dose cited in Table 1. This was set to reduce the risk of bias when an agent is compared to an ineffective dose of a competitor. If more than one dose is evaluated within the same RCT, we will include the results of patients treated with the highest dose in the efficacy comparisons, again ensuring that this dose is within the usual or recommended use of the agent.

**Table 1:** Most commonly used therapies for treatment of diabetic neuropathy

**Class**	**Medication**	**Minimum effective dose (total daily dose)**
Tricyclics	Amitriptyline	50 mg
	Desipramine	100 mg
	Imipramine	100 mg
	Nortriptyline	50 mg
Selective serotonin reuptake inhibitors	Duloxetine	60 mg
	Paroxetine	40 mg
	Venlafaxine^a^	75 mg
Anticonvulsants	Carbamazepine	600 mg
	Gabapentin	900 mg
	Lamotrigine^a^	100 mg
	Oxcarbazepine^a^	600 mg
	Pregabalin	150 mg
	Sodium valproate^a^	400 mg
	Topiramate^a^	100 mg
Topical analgesics	Capsaicin 0.0075%	
	Doxepin^a^	
	Lidocaine 5% patch^a^	
	Mexiletine^a^	
	Pentoxifyline^a^	
Analgesic opiates	Morphine^a^	15 mg
	Oxycodone	20 mg
	Tapentadol^a^	100 mg
	Tramadol	100 mg
Aldose reductase inhibitors^a^	Epalrestat	150 mg
	Ranirestat	20 mg
	Fidarestat	1 mg
	Ponalrestat	600 mg
	Sorbinil	250 mg
Various	Lacosamide^a^	100 mg

^a^No dose recommended by the American Diabetes Association.

### Study design

During the umbrella stage of this systematic review, we will perform a literature search for all systematic reviews (any review with a clear and relevant clinical question, an explicit strategy and list of results) summarizing evidence from randomized trials testing treatments for painful diabetic neuropathy or neuralgia (Additional file 1).

**Figure 2:**
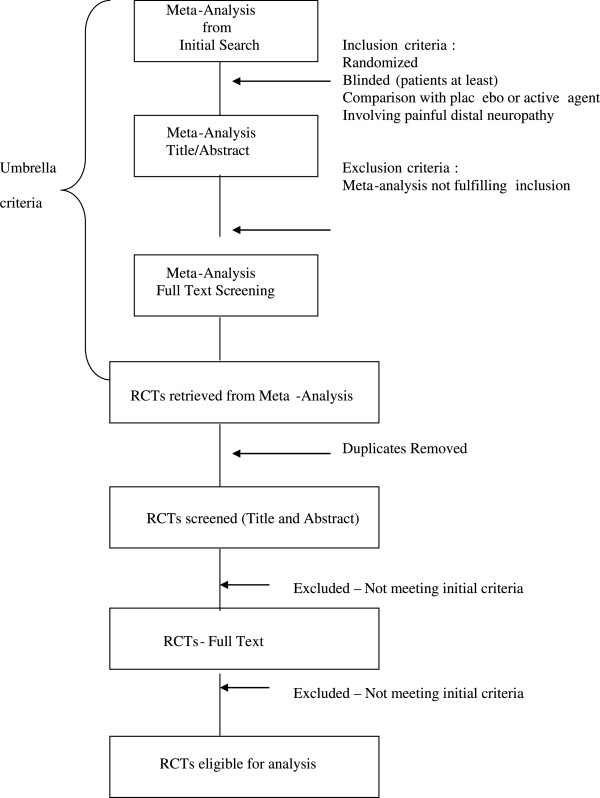
Study selection process.

From these reviews, we will identify eligible trials. These should enroll patients with painful distal symmetrical polyneuropathy and randomly allocate these patients to treatment and control while keeping patients unaware of the therapy to which they have been allocated. There will be no restriction based on language of publication, number of patients or type of diabetes. Figure 2 illustrates the process of study selection.

### Information sources and search methods

We will conduct a comprehensive literature search for systematic reviews published in the last 5 years (2007 to 2011) for diabetic neuropathy or neuralgia in major electronic databases (MEDLINE™ via Ovid, EMBASE via Ovid, and the Cochrane Database of Systematic Reviews). Two study investigators with experience in systematic reviews (VMM and MHM) and an expert reference librarian developed the search strategy (Additional file 1) using a combination of controlled vocabulary (MeSH terms) and key words for the concepts of treatment of neuralgia or diabetic neuropathy.

Two reviewers working independently will identify by title and abstract screening systematic reviews for the treatment of diabetic neuropathy. We will retrieve systematic reviews and meta-analyses deemed to be relevant and review them in full text to extract all eligible RCTs. These will be retrieved in full text for further assessment.

Two reviewers will independently determine RCT eligibility. Disagreement will be solved by consensus, or if consensus is not achieved a third reviewer will serve as an arbitrator. We will assess chance-adjusted agreement (κ statistic) for each step. We will extract descriptive, methodological and analgesic efficacy data in duplicate.

### Outcomes

The main outcome of this study is improvement of pain. We are going to assess this outcome both as a dichotomous (for example, percentage of patients experiencing an improvement of pain of at least ≥30%) and a continuous variable (for example, standardized mean difference on a pain scale). If both dichotomous and continuous outcome data are available from the same trial, data for each variable will be analyzed separately. If pain is reported both in upper and lower extremities, we will analyze data only for the lower extremities. We understand that several outcomes describing pain may be reported and we have therefore decided to build a hierarchy of the pain outcomes we will consider. We will prefer, in decreasing order of relevance, intensity, overall pain, quality, duration, and timing. If pain is reported at multiple time points, we will assess efficacy of treatment at subacute (≤3 months) and chronic timeframes (anything >3 months). If multiple assessments are reported, we will use the longest time point within the aforementioned categories.

**Figure 3:**
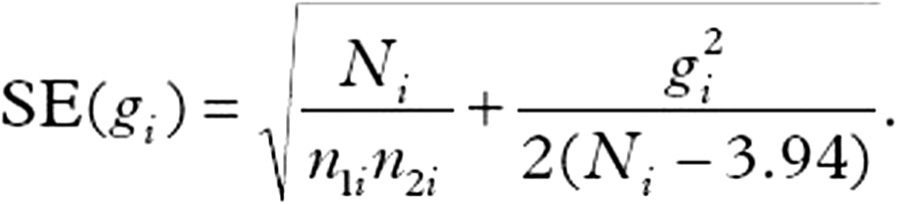
Formula for standard error calculation.

We expect a significant variety of scales measuring our outcomes of interest. We will standardize all scales, hence creating a unitless metric of pain, by expressing differences in number of standard deviations (standardized difference), which will allow comparison among the different scales (Figure 3).

For parallel-arm placebo-controlled trials and comparative effectiveness head-to-head trials, differences will be calculated between arms. Crossover trials are going to be analyzed according to recommendations from the Cochrane Handbook for Systematic Reviews of Interventions [8].

The network approach will generate a ranking list based on efficacy. According to the results we will rank all the included medications from most to least effective based on their effect on pain scores.

### Data collection and extraction

Data from RCTs will be extracted in duplicate, by independent reviewers, using a standardized, piloted, web-based data extraction form. We will re-extract data from each randomized clinical trial on patient demographics, baseline characteristics (duration of diabetes, hemoglobin A_1c_ (HbA_1c_)), study design, sample size, intervention type (class, type and dosages), scale type, reporting of functional status outcomes or impaired sleep pattern.

### Risk of bias assessment

We will use the Cochrane Collaboration’s risk of bias tool to evaluate the methodological quality of RCTs. Two reviewers will independently assess risk of bias using the following domains: random sequence generation (adequate if based on computer generated random numbers, tables of random numbers or similar), allocation concealment (adequate if based on central randomization, sealed envelopes or similar), blinding of patients care givers or outcome assessors, incomplete outcome data (due to high rate of discontinuation, loss to follow-up, type of analysis, or imputation of missing data), selective reporting and other bias (source of funding, whether by not-for-profit or for-profit sources.) Disagreements between reviewers will be resolved by discussion or will be arbitrated by a third reviewer (MHM). We will summarize the risk of bias at all six domains to produce an overall risk of bias for every trial. This will be deemed high if there is presence of high bias in any key domain (allocation concealment or blinding of patients), low if risk of bias is low for all key domains, and unclear in all other cases. We chose a priori to consider allocation concealment and blinding as key quality domains, due to their relative importance for assessment of subjective outcomes such as pain, and have formulated an a priori rule to summarize risk of bias for every class of outcomes across domains, based on scoring of risk of bias in every domain of the Cochrane Risk of Bias tool [8].

### Grading of quality of evidence

We will use the GRADE (‘Grading of Recommendation, Assessment, Development and Evaluation’) framework to evaluate the quality of evidence. Evidence derived from the RCTs will be initially considered high (at low risk of bias) and subsequently downgraded based on methodological limitations, imprecision, inconsistency, indirectness and publication bias [10]. A final generated grade will reflect the confidence we have in the reported estimate of effect.

### Statistical analysis

We will pool odds ratios (ORs) for pain improvement reported as a dichotomous outcome and standardized mean differences (SMDs) using Hedges’ adjusted g for pain scores based on different scales from included studies, accompanying 95% confidence intervals (CI). Then, we will conduct pairwise meta-analyses and estimate the combined efficacy of treatment using a random effects model using the method of DerSimonian and Laird, with the estimate of heterogeneity being taken from the Mantel-Haenszel model [11]. Network meta-analyses will be adopted to combine direct and indirect evidence using Lumley’s generalized linear mixed models based on Frequentists’ method. The Bayesian Markov Chain Monte Carlo method will also be used as an alternative statistical method to validate the results.

### Generalized linear mixed models

We will construct linear mixed models to combine direct and indirect evidence. First introduced by Lumley [12], linear mixed models can be used to estimate the effects of an indirect comparison with two or more linking treatments (paths). For example, to pool the indirect evidence between treatment A and treatment B, we can combine estimates from treatment A to treatment C, treatment B to treatment C, treatment A to treatment D, and treatment B to treatment D. Another advantage of linear mixed models is it can evaluate the agreement of indirect comparisons through different linking treatments, also called ‘incoherence’, and incorporate the incoherence in the calculation of confidence interval of the pooled estimate. If there is direct evidence, linear mixed models also allow us to pool direct and indirect evidence and get more efficient estimate. R statistical software V. 2.15.1 will be used to fit generalized linear mixed models.

### Dealing with missing data

It is likely that some eligible RCTs will not report all relevant data such as the standard deviation or other important variability measures [13]. In order to include these studies in the analysis we will try first to calculate them through algebraic manipulation of the available information such as confidence intervals, p or t values. If that was not possible, we will attempt to contact study authors for these data. If that approach also failed we will borrow these measures from other similar studies as Furukawa *et al*. suggested. When assumptions are made in estimating these measures, such assumptions will be tested in sensitivity analysis [14].

### Subgroup analysis

To investigate potential heterogeneity across studies, we will conduct subgroup analysis based on class of medicine, quality of evidence (low and unclear risk of bias vs high risk of bias), oral versus topical agents, HbA_1c_ level, duration of diabetes, and patients’ age. Random effects meta-regression models will be used to quantify the difference between subgroups and test for statistical significant interactions among subgroups.

### Sensitivity analysis

We will conduct sensitivity analysis to assess the effects of individual studies on the combined estimates and determine whether certain studies dominate the pooled event (particularly if these studies were at high risk of bias). Any imputations made for measures of dispersion (that is, standard deviation) of the difference in pain outcome will be tested in sensitivity analysis. If correlation coefficients are needed to estimate standard deviations, different coefficients will be tested. If certain studies have borderline eligibility status for any reason, analysis will be conducted with and without such studies. Finally, a major sensitivity analysis would be to determine whether Bayesian analysis results are different from the Frequentist method ones. Robustness of analysis (consistency throughout sensitivity analyses and assumptions) would increase our confidence in the comparative effectiveness analysis.

We also plan to use the Bayesian Markov Chain Monte Carlo method to combine direct and indirect evidence and fit the model in WinBUGS V. 1.4.3 [15,16]. This method allows for the pooling of indirect and direct comparisons of effect between the different classes of therapy for diabetic neuropathy, while preserving within-trial randomization, and allowing comparisons of agents not addressed within any of the individual trials [15,16]. A random-effects model will be fitted due to potential for heterogeneity among included trials. The posterior distribution of all parameters will be estimated using non-informative priors, in order for results to be represented solely by the included data. Results will be based from 100,000 iterations, after a 50,000-iteration burn-in. Inconsistency will be evaluated by comparing the estimates from direct comparisons and those from the indirect comparisons for the magnitude and direction of effect. Appropriateness of model fit will be evaluated using the residual deviance, where good model fit is represented by the residual deviance value approximating the number of unconstrained data points [17]. The Bayesian Markov Chain Monte Carlo method will provide us an alternative statistical method to validate our results.

We will use the *I*^2^ statistic and Cochran’s Q test to assess heterogeneity across studies. We will explore heterogeneity for potential intraclass differences.

Finally, publication bias and small study effect will be assessed, whenever possible, using the Egger regression asymmetry test and the Begg adjusted rank correlation test [18,19]. All statistical analyses, except generalized linear mixed models, and the Bayesian Markov Chain Monte Carlo method, will be conducted using STATA V. 12.0.

### Reporting

The study will be reported in accordance with the recommendations set forth by the Preferred Reporting Items for Systematic Reviews and Meta-Analyses (PRISMA) work groups [20]. We will present network graphs depicting the effect size for direct, indirect and combined analyses highlighting the quality and risk of bias in each network loop.

## Discussion

This network meta-analysis aims to synthesize available direct and indirect evidence of effectiveness of analgesics in the treatment of painful diabetic neuropathy. We expect to use evidence of inconsistency between direct and indirect comparisons to identify publication bias affecting the literature on head-to-head comparisons. The network approach will offer the opportunity to generate a ranking based on efficacy. This, along with known side effects, costs, and administration burdens will enable patients and clinicians to make choices that best reflect this evidence, their context, and the informed preferences of patients with painful diabetic neuropathy.

## Competing interests

The authors declare they have no conflicts of interest.

## Authors’ contributions

MLG, AT and JPB developed the search criteria, selection criteria, specified outcome measures determined the data to be abstracted and drafted the manuscript. ZW and OJP determined the analyses and interpretation of data to be conducted. VMM and MHM drafted the manuscript and did critical revisions. All authors read, provided feedback and approved the final manuscript.

## Supplementary Material

Additional file 1 EMBASE/MEDLINE. Click here for file

## References

[B1] Benbow SJ Wallymahmed ME MacFarlane IA Diabetic peripheral neuropathy and quality of life QJM 1998 91 733 737 10.1093/qjmed/91.11.733 10024935

[B2] Wong MC Chung JW Wong TK Effects of treatments for symptoms of painful diabetic neuropathy: systematic review BMJ 2007 335 87 10.1136/bmj.39213.565972.AE 17562735 PMC1914460

[B3] Boulton AJ Pharmacologic management of painful diabetic neuropathy Curr Diab Rep 2008 8 429 430 10.1007/s11892-008-0074-2 18990297

[B4] Bril V England J Franklin GM Backonja M Cohen J Del Toro D Feldman E Iverson DJ Perkins B Russell JW Zochodne D American Academy of Neurology, American Association of Neuromuscular and Electrodiagnostic Medicine, American Academy of Physical Medicine and Rehabilitation Evidence-based guideline: treatment of painful diabetic neuropathy: report of the American Academy of Neurology, the American Association of Neuromuscular and Electrodiagnostic Medicine, and the American Academy of Physical Medicine and Rehabilitation Neurology 2011 76 1758 1765 10.1212/WNL.0b013e3182166ebe 21482920 PMC3100130

[B5] Boulton AJ Pharmacologic management of painful diabetic neuropathy Curr Diab Rep 2008 8 429 430 10.1007/s11892-008-0074-2 18990297

[B6] Lindsay TJ Rodgers BC Savath V Hettinger K Treating diabetic peripheral neuropathic pain Am Fam Physician 2010 82 151 158 20642268

[B7] Boulton AJ Vinik AI Arezzo JC Bril V Feldman EL Freeman R Malik RA Maser RE Sosenko JM Ziegler D Diabetic neuropathies: a statement by the American Diabetes Association Diabetes Care 2005 28 956 962 10.2337/diacare.28.4.956 15793206

[B8] Higgins JPGS Cochrane Handbook for Systematic Reviews of Interventions, Version 5.1.0 http://www.cochrane-handbook.org

[B9] Domecq JP Prutsky G Wang Z Elraiyah T Brito JP Mauck K Lababidi MH Leppin A Fidahusse S Prokop LJ Montori VM Murad MH Drugs commonly associated with weight change: umbrella systematic review and meta-analysis (Protocol) Syst Rev 2012 1 44 10.1186/2046-4053-1-44 23020969 PMC3582551

[B10] Balshem H Helfand M Schünemann HJ Oxman AD Kunz R Brozek J Vist GE Falck-Ytter Y Meerpohl J Norris S Guyatt GH GRADE guidelines: 3. Rating the quality of evidence J Clin Epidemiol 2011 64 401 406 10.1016/j.jclinepi.2010.07.015 21208779

[B11] DerSimonian R Laird N Meta-analysis in clinical trials Control Clin Trials 1986 7 177 188 10.1016/0197-2456(86)90046-2 3802833

[B12] Lumley T Network meta-analysis for indirect treatment comparisons Stat Med 2002 21 2313 2324 10.1002/sim.1201 12210616

[B13] Lambert PC Sutton AJ Burton PR Abrams KR Jones DR How vague is vague? A simulation study of the impact of the use of vague prior distributions in MCMC using WinBUGS Stat Med 2005 24 2401 2428 10.1002/sim.2112 16015676

[B14] Furukawa TA Barbui C Cipriani A Brambilla P Watanabe N Imputing missing standard deviations in meta-analyses can provide accurate results J Clin Epidemiol 2006 59 7 10 10.1016/j.jclinepi.2005.06.006 16360555

[B15] Lu G Ades AE Combination of direct and indirect evidence in mixed treatment comparisons Stat Med 2004 23 3105 3124 10.1002/sim.1875 15449338

[B16] Salanti G Higgins JP Ades AE Ioannidis JP Evaluation of networks of randomized trials Stat Methods Med Res 2008 17 279 301 17925316 10.1177/0962280207080643

[B17] Caldwell DM Welton NJ Ades AE Mixed treatment comparison analysis provides internally coherent treatment effect estimates based on overviews of reviews and can reveal inconsistency J Clin Epidemiol 2010 63 875 882 10.1016/j.jclinepi.2009.08.025 20080027

[B18] Begg CB Mazumdar M Operating characteristics of a rank correlation test for publication bias Biometrics 1994 50 1088 1101 10.2307/2533446 7786990

[B19] Egger M Davey Smith G Schneider M Minder C Bias in meta-analysis detected by a simple, graphical test BMJ 1997 315 629 634 10.1136/bmj.315.7109.629 9310563 PMC2127453

[B20] Liberati A Altman DG Tetzlaff J Mulrow C Gotzsche PC Ioannidis JP Clarke M Devereaux PJ Kleijnen J Moher D The PRISMA statement for reporting systematic reviews and meta-analyses of studies that evaluate health care interventions: explanation and elaboration J Clin Epidemiol 2009 62 e1 34 10.1016/j.jclinepi.2009.06.006 19631507

