# Supplementary material for: Pharmacologic interventions for painful diabetic neuropathy: an umbrella systematic review and comparative effectiveness network meta-analysis (Protocol)
Source: Syst Rev. 2012 Dec 2;1:61. doi: 10.1186/2046-4053-1-61 (PMC3534585; doi:10.1186/2046-4053-1-61)
Supplement: Additional file 1 — EMBASE/MEDLINE. [file 2046-4053-1-61-S1.doc]

# Appendix 1

EMBASE/MEDLINE

1 exp Diabetic Neuropathies/

2 limit 1 to meta analysis

3 systematic review.mp.

4 1 and 3

5 2 or 4

6 meta analysis/

7 exp "systematic review"/

8 1 and (6 or 7)

9 4 or 8

10 remove duplicates from 9

11 limit 10 to (editorial or letter)

12 10 not 11

13 exp Neuralgia/

14 limit 13 to meta analysis

15 3 and 14

16 exp neuropathic pain/

17 16 and (6 or 7)

18 3 and 13

19 from 18 keep 752-804

20 from 14 keep 404-467

21 17 or 19 or 20

22 remove duplicates from 21

23 limit 22 to (editorial or letter)

24 22 not 23

25 limit 24 to yr="2005 -Current"
